# Unveiling the Diagnosis of Pediatric Dizziness in a Tertiary Care Hospital: The Complementary Role of Vestibular and Neurological Evaluations

**DOI:** 10.1055/s-0044-1801318

**Published:** 2025-04-22

**Authors:** Ahmed Khater, Wafaa Samir Mohamed, Diana Hanna, Yostina Adel Abdelmalak, Nahla Gad

**Affiliations:** 1 Audiovestibular Medicine Unit, Department of Otorhinolaryngology, Faculty of Medicine, Zagazig University, Zagazig, Egypt; 2 Department of Neurology, Faculty of Medicine, Zagazig University, Zagazig, Egypt; 3 Department of Pediatrics, Faculty of Medicine, Zagazig University, Zagazig, Egypt

**Keywords:** dizziness, pediatric, migraine, vestibular, vertigo

## Abstract

**Introduction** Pediatric dizziness is not a rare symptom, and it has a significant impact on the child's psychophysical wellbeing and quality of life. There are diverse etiologies of dizziness in children; however, it is challenging to diagnose. Vestibular and neurological assessments are crucial in the diagnosis of pediatric dizziness.

**Objective** To outline the most common etiologies of dizziness in children and to investigate the complementary role of the vestibular and neurological evaluations in the assessment of dizzy children.

**Methods** We conducted a case-control study including 40 children with a complaint of dizziness and 40 healthy children as the control group. We assessed their full medical history audiovestibular function through pure tone audiometry, videonystagmography examination, cervical vestibular evoked myogenic potentials, the results of video head impulse tests, as well as their electroencephalograms and brain magnetic resonance imaging scans.

**Results** The mean age of the 40 children who were presented with dizziness was of 13.65 years. Migraine was found to be the commonest cause of vertigo (27.5%), while benign paroxysmal vertigo of childhood was detected in 17.5%, and central nervous system causes, in 12.5%. The diagnosis could not be ascertained in 9 (22.5%) patients.

**Conclusion** The diagnosis of the etiologies of pediatric dizziness is challenging; however, detailed medical history, a comprehensive examination, a multidisciplinary approach, along with full vestibular and neurological assessments, are essential to reach an accurate diagnosis.

## Introduction

Dizziness is a common complaint that include numerous symptoms, such as unsteadiness, clumsiness, lightheadedness, loss of balance, and vertigo. 1 Vestibular disorders represent the commonest causes of pediatric dizziness, with a reported prevalence ranging from 0.5% to 15%. 2 3 4 Nevertheless, the actual incidence of dizziness in children is still underestimated, as the expressions of disequilibrium are usually attributed to motor incoordination or behavior, not to mention the fact that children have difficulty verbalizing their symptoms. 5 Dizziness is a challenging symptom to define and diagnose, and it is also difficult to measure and manage. 6 Due to the recognized effect of vertigo on the psychophysical wellbeing and health related quality of life of children, its management requires a multidisciplinary approach. 7 There are diverse etiologies of vertigo in children, and the commonest type is migraine-related vertigo, which is frequently associated with a positive familial history of migraine with the progression to classic migraine later on. 8 9 In vestibular migraine, the vestibular symptoms are sometimes associated with headache. 8 10 Other neurological causes include epileptic, neoplastic, vascular and several other causes. 4 11 Vestibular testing in dizzy children is crucial, but it is not easy to perform. 12

The present study was conducted to outline the leading causes of dizziness in children and to investigate the complementary role of vestibular and neurological evaluations in the assessment of dizzy children.

## Methods

The present case-control study was conducted at the Audiovestibular Medicine Unit, Department of Otorhinolaryngology, in collaboration with the Departments of Neurology and Pediatrics of the Faculty of Medicine of Zagazig University, Zagazig, Egypt. It included 40 children presenting with a complaint of dizziness and 40 healthy controls from March 2023 to Jan 2024.

The Institutional Review Board (IRB) approved the protocol under no. ZU-IRB #10487, and written informed consent forms were signed by the legal guardians of the participants before enrollment.

All dizzy children and adolescents of both sexes, aged between 10 and 18 years, who were presented with unsteadiness, imbalance, clumsiness, light-headedness, or vertigo age were enrolled. Children who had any otorhinolaryngological disease, history of neurological disorders or any systemic illness that affected the vestibular system, head trauma, behavioral and/or genetic syndromes, as well as those who received ototoxic medications were excluded from the study.

Full medical history, including a detailed dizziness history and family history of migraine, was taken. A complete otoneurological examination as well as oculomotor and musculoskeletal examinations were performed. We also assessed electroencephalograms (EEG) and brain magnetic resonance imaging (MRI) scans and conducted An audiovestibular evaluation through pure tone audiometry (PTA), speech audiometry, immittancemetry, videonystagmography (VNG), an assessment of the cervical vestibular evoked myogenic potentials (cVEMP), and video head impulse tests (VHIT).

The diagnostic causes of dizziness, such as vestibular migraine (VM), benign paroxysmal vertigo of childhood BPVC, Ménière's disease, and others, were established based on the standard criteria contained in the published literature. 13 14 15 16

## Audiovestibular Evaluation

1. **PTA:** The Air conduction thresholds were tested at frequencies ranging from 0.25 kHz to 8 kHz frequencies, and THE bone conduction thresholds at frequencies ranging from 0.5 kHz to 4.0 kHz using a two-channel audiometer (Amplaid 311, Amplifon SpA, Milan, Italy).
2. **Speech audiometry:** It included speech recognition threshold (SRT) testing and the word recognition score (WRS).
3. **Immittancemetry:** Acoustic reﬂex studies and tympanometry were elicited ipsilaterally and contralaterally at the frequencies of 0.5 kHz, 1 kHz, 2 kHz, and 4 kHz using and immittancemeter (MADSEN Zodiac 901, Otometrics, Taastrup, Denmark).
4. **VNG test battery:** We used the Ulmer VNG (Inventis, Padua, Italy), version 3.4.0.38, which includes infrared goggles, which can be used to assess spontaneous nystagmus and conduct oculomotor tests (saccade, smooth pursuit, gaze, optokinetic), positional tests, positioning tests (Dix-Hallpike and the roll test), and caloric testing.
4.1 **Bithermal caloric test:** It was conducted with the child in the supine position, with the head elevated to 30°. Cold and warm water irrigation were applied to both ears for 30 seconds at temperatures of 30°C 44°C respectively. 17

### Identification of the Affected Side

- By comparing one side with the other, the unilateral weakness (UW) can be calculated and expressed as a percentage as follows:

- An asymmetric response to a symmetric stimulation defines the directional preponderance (DP). The absolute DP represents, in a given direction, a calculation of the preponderance of the nystagmus intensity, expressed as velocity (degree per second) rather than a percentage.

### Interpretation of the Caloric Testing

The test is deemed abnormal when there is canal paresis > 25% or DP > 30% as per the Jongkees formula. 18

**5. Cervical vestibular evoked myogenic potentials (cVEMP) measurements:** Recordings were carried out using the ICS charter EP 200 system (Otometrics). A tone burst stimulus of 500 Hz was transported to the tested ear at an intensity of 95 dB nHL. The analysis time for each response was 50 ms, while the average for each run was of 150 sweeps.
**6. Video head impulse test (VHIT):** We used an EYE SEE CAM VHIT, interacoustics impulse system (Interacoustics, Middelfart, Denmark). Recordings were made for each of the six semicircular canals (horizontal, left anterior–right posterior [LARP], and right anterior–left posterior [RALP]).

## Measured Parameters

**Gain**

The abnormal results were of two types: normal gain along with refixation saccades and low gain (< 0.6) either with or without saccades.

### Catch-Up Saccade

Catch-up saccades that occurred after head impulses were considered overt saccades, while those that occurred during head impulses were considered covert saccades

#### Statistical Analysis

The data were analyzed using the IBM SPSS Statistics for Windows (IBM Corp., Armonk, NY, United States) software, version 26.0. The qualitative data were expressed as numbers and percentages, and the quantitative parametric data, as mean ± standard deviation values. The Chi-squared (χ ^2^ ) test was used for the categorical data. The student's *t* -test was used for normally distributed variables, while the Man-Whitney U test was used for the non-parametric variables. Correlation was calculated using the Pearson correlation coefficient. Values of *p* ≤ 0.05 were considered statistically significant.

## Results

We enrolled 40 (22 male and 18 female) children who presented with a complaint of dizziness, a with mean age of 13.65(± 2.64) years and a mean duration of the illness of 13.4 (± 13.27) months. The duration of the vertigo attacks, the type of vertigo complaint, the aggravating and relieving factors, as well as the associated symptoms are shown in Table 1 : 45% of the cases presented mainly a complaint of imbalance, the attack occurred spontaneously in half of the patients, rest was the most common relieving factor (in 90% of the cases), nausea and vomiting were experienced by 45% of the patients as associated symptoms, and overlapping symptoms occurred in some patients.

**Table 1:** History of present illness among the patient group

Variable		Patients (N = 40)	
**Duration of illness (months)**			
Mean ± standard deviation		13.40 ± 13.27	
Median (interquartile range)		9 (4–21)	
		**n**	**%**
**Duration of attack** **(minutes)**	≥ 1	8	20
	2	2	5
	5	4	10
	10	6	15
	15	4	10
	30	6	15
	60	2	5
	> 60	8	20
**Nature of complaint**	Imbalance	18	45
	Rotation of surroundings	8	20
	Self-rotation	14	35
**Aggravating factor**	Spontaneous	20	50
	Stress	10	25
	Riding in vehicles	4	10
	Rolling in bed	4	10
	Sudden head movements	4	10
	Leaning forward	4	10
	Loud sounds	2	5
**Relieving factor**	Rest	36	90
	Analgesics	8	20
	Medication (depakin)	2	5
**Associated symptoms**	Generalized headache	20	50
	Nausea, vomiting	18	45
	Phono/photophobia	18	45
	Temporal headache	10	25

The audiological evaluation through PTA revealed that 2 (5%) patients with unilateral left-sided sensorineural hearing loss (SNHL): 1 was diagnosed with Ménière's disease and the other presented otolith dysfunction. Moreover, statistically significant differences were detected between patients and controls at the frequencies of 0.25 KHz ( *p* = 0.013) and 8 KHz ( *p* < 0.001) in the right ear, and at 4 KHz ( *p* = 0.15) in the left ear. The SRT and WRS were tested to confirm the outcomes of the PTA, and no statistically significant differences were found between patients and controls ( *p* > 0.05) Table 2 .

**Table 2:** Comparison between patients and controls regarding PTA, SRT, and WRS

Variable		Patients (N = 40): mean ± SD	Controls (N = 40) : mean ± SD	*p*
**250 Hz**	**Right**	13 ± 3.77	11.25 ± 2.22	0.013*
	**Left**	13.25 ± 9.07	11.25 ± 2.22	0.179
**500 Hz**	**Right**	11.25 ± 3.58	11.5 ± 2.35	0.713
	**Left**	13 ± 9.38	11.5 ± 2.35	0.329
**1 KHz**	**Right**	11.25 ± 2.55	11.51 ± 2.34	0.636
	**Left**	12.75 ± 10.32	11.51 ± 2.34	0.460
**2 KHz**	**Right**	11 ± 1.62	11.49 ± 2.3	0.274
	**Left**	13.5 ± 12.47	11.49 ± 2.3	0.319
**4 KHz**	**Right**	9.5 ± 2.76	9 ± 2.05	0.360
	**Left**	12.5 ± 8.66	9 ± 2.05	0.015*
**8 KHz**	**Right**	12.5 ± 2.56	9 ± 2.08	< 0.001*
	**Left**	12 ± 7.33	9.75 ± 2.08	0.065
**SRT**	**Right**	10.75 ± 2.81	11.5 ± 2.34	0.198
	**Left**	12.25 ± 9.52	11.51 ± 2.34	0.634
**WRS (%)**	**Right**	99.8 ± 2.04	99.44 ± 1.4	0.360
	**Left**	95.8 ± 15.11	99.4 ± 1.47	0.137

**Abbreviations:** PTA, pure tone audiometry; SD, standard deviation; SRT, speech recognition threshold; WRS, word recognition score.

**Note:** *Statistically significant.

Regarding the VNG assessment, abnormal results on the smooth pursuit and optokinetic (OPK) eye movement tests were found in 4 (10%) patients. Moreover, we detected a significantly higher latency ( *p* < 0.001) and lower velocity ( *p* < 0.001) among dizzy children compared with the controls bilaterally, as well as higher slow-phase velocity (SPV) than that of the control group ( *p* = 0.003) on the OPK test in the left eye. The results of the caloric test were abnormal in 2 (5%) children, and we observed a significantly higher UW and DP among the patients compared to the controls ( *p* < 0.001) Table 3 .

**Table 3:** Comparison between patients and controls regarding the VNG tests

Saccade		Patients (N = 40): mean ± SD	Controls (N = 40): mean ± SD	*p*
**Right**	**Latency**	281.4 ± 42.4	243.4 ± 29.52	< 0.001*
	**Velocity (%)**	308.2 ± 71.67	388.55 ± 70.98	< 0.001*
	**Accuracy (%)**	91.05 ± 7.86	93.4 ± 5.83	0.132
**left**	**Latency**	276.5 ± 39.74	248.9 ± 23.89	< 0.001*
	**Velocity (%)**	309.05 ± 53.45	362.05 ± 60.82	< 0.001*
	**Accuracy (%)**	92.55 ± 9.07	94.35 ± 5.58	0.288
**Smooth pursuit eye movement**				
**Right**	**0.3 Hz**	0.86 ± 0.09	0.83 ± 0.08	0.119
	**0.45 Hz**	0.81 ± 0.11	0.82 ± 0.09	0.657
**Left**	**0.3 Hz**	0.84 ± 0.09	0.82 ± 0.07	0.270
	**0.45 Hz**	0.8 ± 0.1	0.83 ± 0.08	0.142
**Optokinetic eye movement**				
**Right**	**Gain**	0.81 ± 0.11	1.33 ± 1.9	0.087
	**SPV**	14.42 ± 1.74	14.38 ± 2.19	0.928
**left**	**Gain**	1.01 ± 1.65	1.29 ± 1.68	0.454
	**SPV**	13.59 ± 3.04	11.87 ± 1.89	0.003*
**Caloric test**				
**Right**	**Cold**	15.85 ± 8.77	20.63 ± 6.31	0.006*
	**Warm**	14.39 ± 9.23	20.47 ± 7.02	0.001*
**Left**	**Cold**	14.91 ± 10.61	20.89 ± 7.68	0.005*
	**Warm**	16.95 ± 8.69	20.38 ± 7.18	0.057
**Unilateral Weakness (%)**		12.40 ± 7.563	4.70 ± 1.949	< 0.001*
**Directional preponderance (%)**		15.55 ± 7.79	7.8 ± 2.46	< 0.001*

**Abbreviations:** SD, standard deviation; SPV, slow-phase velocity; VNG: videonystagmography.

**Note:** *Statistically significant.

As for the cVEMP testing, 4 (10%) cases were detected with abnormalities (2 patients showed an asymmetry ratio at 500 Hz, and 2 showed an asymmetry ratio at 500 Hz and 1 KHz). The patients presented a statistically significant lower P1-N1 amplitude at 0.5 KHz and 1 KHz bilaterally, lower P1 latency at 0.5 KHz in the left ear, lower N1 latency at 1 KHz in the right ear, as well as a statistically significant higher asymmetry ratio at 0.5 KHz and 1 KHz in comparison to the controls. We also detected 2 (5%) patients with abnormal low gain VHIT at the left side, as well as statistically significant differences between cases and controls regarding the VHIT results in both ears ( *p* < 0.001) with lower mean values among the patients Table 4 .

**Table 4:** Comparison between patients and controls regarding the cVEMP and VHIT

cVEMP		Patients (N = 40): mean ± SD	Controls (N = 40): mean ± SD	*p*
**P1-N1 amplitude**				
**Right**	**500 Hz**	205.98 ± 139.67	314.8 ± 188.62	0.004*
	**1 KHz**	153.27 ± 98.23	211.38 ± 126.4	0.024*
**Left**	**500 Hz**	231.5 ± 134.14	313.53 ± 203.37	0.036*
	**1 KHz**	143.71 ± 77.51	207.25 ± 134.91	0.011*
**P1 latency**				
**Right**	**500 Hz**	14.86 ± 1.65	15.52 ± 1.92	0.103
	**1 KHz**	13.46 ± 1.34	14.23 ± 2.13	0.056
**Left**	**500 Hz**	14.67 ± 1.14	15.42 ± 1.83	0.03*
	**1 KHz**	13.8 ± 1.64	14.51 ± 2.36	0.122
**N1 latency**				
**Right**	**500 Hz**	23.05 ± 2.04	23.96 ± 2.5	0.078
	**1 KHz**	20.68 ± 2.36	22.61 ± 3	0.002*
**Left**	**500 Hz**	23.07 ± 2.19	23.78 ± 2.54	0.184
	**1 KHz**	21.46 ± 1.82	22.35 ± 4.05	0.208
**Asymmetry ratio (%)**				
	**500 Hz**	15.22 ± 9.33	5.57 ± 4.45	< 0.001*
	**1 KHz**	12.87 ± 10.47	6.54 ± 5.55	0.001*
**VHIT**				
**Right**	**Lateral**	0.95 ± 0.22	1.13 ± 0.15	< 0.001*
	**Posterior**	0.94 ± 0.23	1.12 ± 0.15	< 0.001*
	**Anterior**	1 ± 0.22	1.19 ± 0.19	< 0.001*
**Left**	**Lateral**	0.9 ± 0.23	1.16 ± 0.18	< 0.001*
	**Anterior**	0.93 ± 0.2	1.21 ± 0.15	< 0.001*
	**Posterior**	1.01 ± 0.31	1.17 ± 0.19	0.006*

**Abbreviations:** cVEMP, cervical vestibular evoked myogenic potentials; VHIT, video head impulse test.

**Note:** *Statistically significant.

In terms of the EEGs, 3 (7.5%) patients presented abnormalities: 2 had bitemporal epileptic discharge, and 1, frontal epilepsy. The MRI scans showed that 2 (5%) patients presented abnormalities: left-sided mesial temporal sclerosis in 1 case, and bilateral vestibular schwannoma in the other case.

**Fig. 1:**
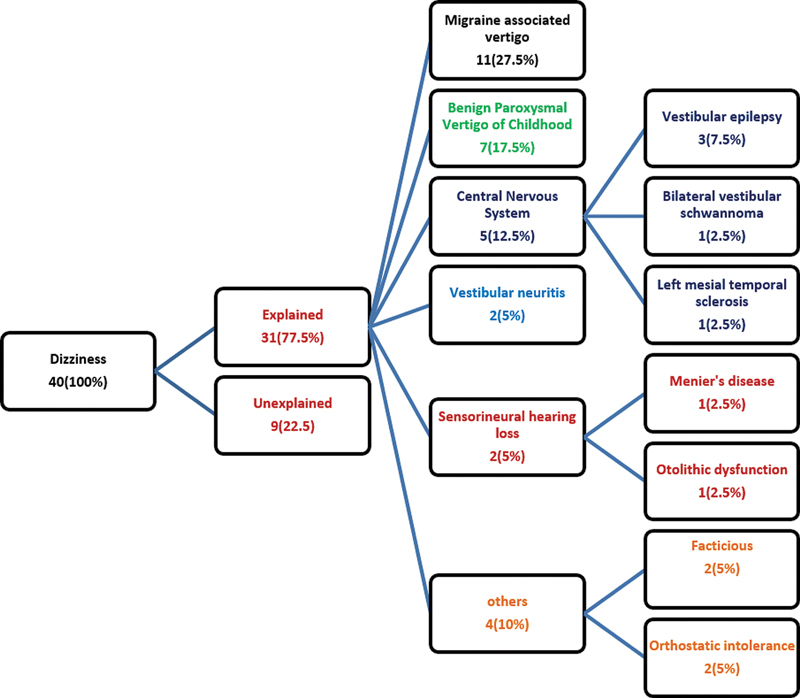
Algorithm presenting the diagnosis of dizziness among the studied group.

The diagnoses of the patients are presented in Fig. 1 ; the most common cause was migraine-associated vertigo in 11 (27.5%) cases; however, the diagnosis could not be ascertained in 9 (22.5%) patients.

## Discussion

Differences have been reported 12 19 between pediatric and adult patients regarding the underlying cause of dizziness; benign paroxysmal positional vertigo (BPPV), Ménière's disease, and vestibular neuritis are considered the commonest causes in adults, while VM and BPVC are the commonest in children. The present study showed that the commonest cause of dizziness was VM, in 11 (27.5%) patients, as per the International Classification of Headache Disorders, third edition (ICHD-3). 13 This finding is in line with those of previous studies. 20 21 The presentation of migraine differs between children and adults, with children presenting shorter headache episodes that tend to occur bilaterally. 14 It is worth mentioning that concomitant headache is not essential for the diagnosis of VM if other symptoms such as visual aura, nausea, photophobia, and phonophobia occur during the dizziness attack. Recent evidence 22 proposes that VM is physiologically the same as other forms of migraine, but central and/or peripheral vestibular system occurs during episodes, mainly through the trigeminovascular pathway.

The second most common cause of dizziness identified in the present study was BPVC (in 17.5% of the patients). In agreement with our findings, several authors 9 23 24 have identified BPVC as the second most common cause of vertigo after migraine in children and adolescents. On the other hand, several studies 25 26 have reported BPVC as the commonest cause of pediatric dizziness. This can be explained by the fact that the incidence differs according to the age group. Among pediatric patients, BPVC was experienced the most in preschool children, and VM afterwards; however, BPVC and VM became equally common in the school age group. In adolescents, VM has been reported to be the most common cause. 27 Notably, the etiology of BPVC is thought to be due to the disruption of blood flow to the brain 28 (the “vascular hypothesis”), and VM and BPVC present a similar etiology. 29 Moreover, 8/18 (44.4%) of the patients diagnosed with VM and BPVC in the present study had a first-degree relative with a history of migraine. It has also been reported that many children with BPVC may develop migraine later in life. 30 31

Causes linked to the central nervous system were detected in 5 (12.5%) patients in the current study: vestibular epilepsy was identified in 3 (7.5%) subjects. Haripriya et al. 25 found an incidence of 8/89 (9%) of vestibular epilepsy, and other studies reported lower incidences. 4 23 24 32

In the present study, one patient had the diagnosis of bilateral vestibular schwannoma and another, of left mesial temporal sclerosis. Disorders of the CNS can create an imbalance caused by injuries to the brainstem, cerebellum and the thalamus or the cortex, and they can occur with vestibular peripheral complications. 33

Regarding the vestibular tests, we noted that the caloric testing was difficult and challenging to perform, even though we included older children (aged between 10 and 18 years): it revealed abnormalities in 2 (5%) patients; the same percent was detected by the VHIT, and those patients were diagnosed with vestibular neuritis. It has been reported 34 that VHIT has advantages compared to caloric tests because it assesses all six semicircular canals individually and is better tolerated by children. Nevertheless, it has a low sensitivity for well-compensated or mild vestibular loss. 34

Choung et al. 23 detected vestibular neuritis in 2% out of 52 dizzy patients. A higher incidence (14%) was reported by Ravid et al. 24

It is worth mentioning that the audiovestibular testing conducted in the present study revealed abnormalities in a limited percentage of our patients (10%); however, we detected statistically significant differences between patients and controls regarding the different tests conducted, which reveals a trend towards abnormality among our patients, but not fulfilling the criteria to be considered abnormal. This highlights the need for more case-control studies with larger samples and extended duration, with comprehensive assessments and reassessments to accurately diagnose children and adolescents.

In the current study, 1 (2.5%) patient was diagnosed with Ménière's disease. History, PTA, VNG, and cVEMP were all necessary to establish the diagnosis. Elghafar et al. 32 reported only 3 cases (0.35%) of Ménière's disease. Consistent with these findings, Ravid et al. 24 did not detect any cases of Ménière's disease in 62 subjects, and Balatsouras et al. 9 detected 1 case out of 50 patients.

We found 1 (2.5%) patient with otolith dysfunction with unilateral SNHL and abnormal vestibular tests. Haripryia et al. 25 reported 4 (4.5%) children with otolith dysfunction.

Other causes, such as factitious vertigo (2; 5%) and orthostatic intolerance (2; 5%) were detected in the current study. Regarding factitious dizziness, our results were consistent with those of Haripriya et al., 25 who detected a rate of 5.6% of children who feigned dizziness to avoid exams or punishment etc. These children have been evaluated but remained undiagnosed, and they termed this *factitious dizziness* . Upon diagnosis, all children admitted they had feigned dizziness. Notably, other studies have described the term *psychogenic* vertigo, 21 24 35 but, in the current study, no underlying psychiatric illness was identified in the 2 (5%) children with factitious vertigo.

Orthostatic intolerance has recently been reported in pediatric patients. 36 37 The typical presentations involve a sense of imminent fainting, decreased concentration, lightheadedness, blurred vision, headache, nausea, abdominal pain, as well as pallor, diaphoresis, tachycardia, bradycardia or hypotension. 25

We could not reach a precise diagnosis in 9 (22.5%) children, which is in line with the study by Elghaffar et al., 32 who reported a rate of 20.5% of dizzy children who were undiagnosed. Other studies have reported unclassified etiology in 13 to 18% of the subjects. 23 38 Such patients need more follow-up than those with a specific diagnosis. Interestingly, some children experience a spontaneous resolution of the vertigo symptoms, 39 which may contribute to the unclassified cause in 43.6% of them. 7

## Conclusion

Dizziness constitutes a great diagnostic challenge, especially in the pediatric age group. In the current study, the most common cause of dizziness in children and adolescents was VM, followed by BPVC. Detailed history, otological examination, audiovestibular assessments, as well as a multidisciplinary approach, including a neurological evaluation, are all crucial to establish a diagnosis. However, in many children, dizziness remains undiagnosed. Future case-control studies with larger samples and longer duration are still required, with comprehensive audiovestibular assessments and reassessments, to better standardize the values found and detect the disease in its early stages among children and adolescents.

## Limitations

The main limitation of the current study is the small sample size. Moreover, we did not investigate the proper management and follow-up of the patients. This will be considered a point for our future research.
